# High ubiquitin‐specific protease 44 expression induces DNA aneuploidy and provides independent prognostic information in gastric cancer

**DOI:** 10.1002/cam4.1090

**Published:** 2017-05-23

**Authors:** Sho Nishimura, Eiji Oki, Koji Ando, Makoto Iimori, Yu Nakaji, Yuichiro Nakashima, Hiroshi Saeki, Yoshinao Oda, Yoshihiko Maehara

**Affiliations:** ^1^ Department of Surgery and ScienceGraduate School of Medical SciencesKyushu UniversityFukuokaJapan; ^2^ Department of Molecular OncologyGraduate School of Medical SciencesKyushu UniversityFukuokaJapan; ^3^ Department of Anatomic PathologyPathological SciencesGraduate School of Medical SciencesKyushu UniversityFukuokaJapan

**Keywords:** Chromosome instability, gastric cancer, prognosis, USP44

## Abstract

Chromosomal instability (CIN), characterized by aneuploidy, is a major molecular subtype of gastric cancer. The deubiquitinase USP44 is an important regulator of APC activation in the spindle checkpoint and leads to proper chromosome separation to prevent aneuploidy. Aberrant expression of USP44 leads CIN in cells; however, the correlation between USP44 and DNA aneuploidy in gastric cancer is largely unknown. We analyzed USP44 expression in 207 patients with gastric cancer by immunohistochemistry and found that the proportion of USP44 expression was higher in gastric cancer tumors (mean, 39.6%) than in gastric normal mucosa (mean, 14.6%) (*P* < 0.0001). DNA aneuploidy was observed in 124 gastric cancer cases and high USP44 expression in cancer strongly correlated with DNA aneuploidy (*P* = 0.0005). The overall survival was significantly poorer in the high USP44 expression group compared with the low USP44 group (*P* = 0.033). Notably, USP44 expression had no prognostic impact in the diploid subgroup; however, high USP44 expression was a strong poor prognostic factor for progression‐free survival (*P* = 0.018) and overall survival (*P* = 0.036) in the aneuploid subgroup. We also confirmed that stable overexpression of USP44 induced somatic copy‐number aberrations in hTERT‐RPE‐1 cells (50.6%) in comparison with controls (6.6%) (*P* < 0.0001). Collectively, our data show USP44 has clinical impact on the induction of DNA aneuploidy and poor prognosis in the CIN gastric cancer subtype.

## Introduction

Gastric cancer was the third most common cause of cancer‐related death in the world in 2012 1. Several classification systems have been proposed for gastric cancer, such as the Lauren classification and WHO classification, based on histopathological findings 2. These classification systems are useful for characterizing the cancer malignancy for each patient, but have little significance for determining the treatment plan and prognosis. Several treatment strategies for gastric cancer are currently in use, including chemotherapy, molecular targeting treatment, and immunotherapy; however, more precise molecular classification and determination of clinical markers in gastric cancer are required to improve treatment effectiveness 3. In 2014 The Cancer Genome Atlas (TCGA) Research Network classified gastric adenocarcinoma into four molecular subtype groups based on genomic database analysis: (i) Epstein–Barr virus‐associated DNA hypermethylation, (ii) microsatellite instability (MSI) high status, (iii) genomically stable (GS), and (iv) chromosomal instability (CIN) 1. The CIN group represents the largest subtype of gastric cancer (49.8%) and this group is defined by DNA aneuploidy 1.

CIN is a hallmark of cancer cells, and aneuploidy is a main characteristic of CIN 4. Aneuploidy is the presence of an abnormal number of chromosomes in cells 5 and is found in the majority (70–90%) of cancer cells 6. While aneuploidy is a common characteristic of cancer cells, its role in tumor initiation and progression is unclear. Several studies have suggested that aneuploidy has tumor‐promoting or tumor‐suppressive contributions in cancer development 7.

Deubiquitinating enzymes are members of the protease family that cut the isopeptide bond between ubiquitin and ubiquitin or between ubiquitin and the target protein 8. To date, approximately 100 deubiquitinating enzymes have been reported 9. These enzymes regulate critical cellular pathways, such as cell proliferation, cell signal transmission, DNA damage repair and more 10. Ubiquitin‐specific protease 44 (USP44) is a deubiquitinating enzyme that belongs to the ubiquitin‐specific protease (USP) family. During mitosis, USP44 prevents the premature activation of the anaphase‐promoting complex (APC) by stabilizing the APC‐inhibitory Mad2–Cdc20 complex in spindle assembly checkpoint (SAC) 11. USP44 also acts independently by regulating centrosome separation, positioning, and mitotic spindle geometry 12. These functions support proper chromosome separation and prevent CIN, including aneuploidy. Although USP44 suppression and overexpression have been correlated with malignancy 12, 13, 14, 15, 16, the correlation between USP44 overexpression and DNA aneuploidy is largely unknown.

In this study, we examined the relationship between USP44 overexpression and DNA aneuploidy using clinical specimens of gastric cancer and provide the first evidence for the prognostic significance of evaluating USP44 expression combined with DNA aneuploidy.

## Material and Methods

### Patients

This study included 207 Japanese patients with primary gastric cancer, all of who underwent a gastrectomy between 1994 and 2006 at the Department of Surgery and Science, Graduate School of Medical Sciences, Kyushu University Hospital, Fukuoka. The patient group included 138 men and 69 women, ranging in age from 29 to 90 years (mean, 63.7 years). Each patient provided informed consent, and patients who refused consent were not included. Patients who were treated preoperatively with cytotoxic drugs were not included in this study. We completely resected for primary gastric cancer in all cases excepted for Stage IV cases in this study. In addition, we performed appropriate treatment as required for each case after operation.

### Tumor staging

A thorough histological examination was performed using hematoxylin and eosin‐stained tissue preparations, and the histological classification was made according to the general rules of the 14th edition of Japanese Classification of Gastric Carcinoma set by the Japanese Gastric Cancer Association 17. Depth of invasion and lymph node metastasis were determined by pathological examination of surgically resected specimens. Distant metastases were determined by preoperative images, intraoperative findings and postoperative examination. Postoperative pathological T (pT), N (pN), M (pM), and Stage (pStage) were used for all cases.

### Immunohistochemical staining

Formalin‐fixed, paraffin‐embedded tissue specimens were used for immunohistochemical staining. A paraffin block that contained both cancerous tissue, invading the deepest area of the stomach wall, and adjacent noncancerous tissue was used in each case. Briefly, the sections were pretreated with autoclaving (121°C) for 15 min in 0.01 mol/L citrate‐buffered saline (pH 9.0) for antigen retrieval. Endogenous peroxidase activity was blocked by incubation with 3% H_2_O_2_ for 30 min at room temperature. Nonspecific reaction was blocked by 10% goat normal serum for 10 min at room temperature. The sections were incubated with USP44 mouse monoclonal antibody (Clone 1F9, 1:150; OriGene Technologies, Inc., Rockville, MD 20850) at 4°C overnight. Streptavidin‐biotin complex and horseradish peroxidase were applied, and the reaction products were visualized using the Histofine SAB‐PO (M) immunohistochemical staining kit (Nichirei, Tokyo, Japan), according to the manufacturer's instructions. Two blinded observers (S. N. and Y. N.) independently examined immunostained sections.

### Evaluation of USP44 expression

**Figure 1:**
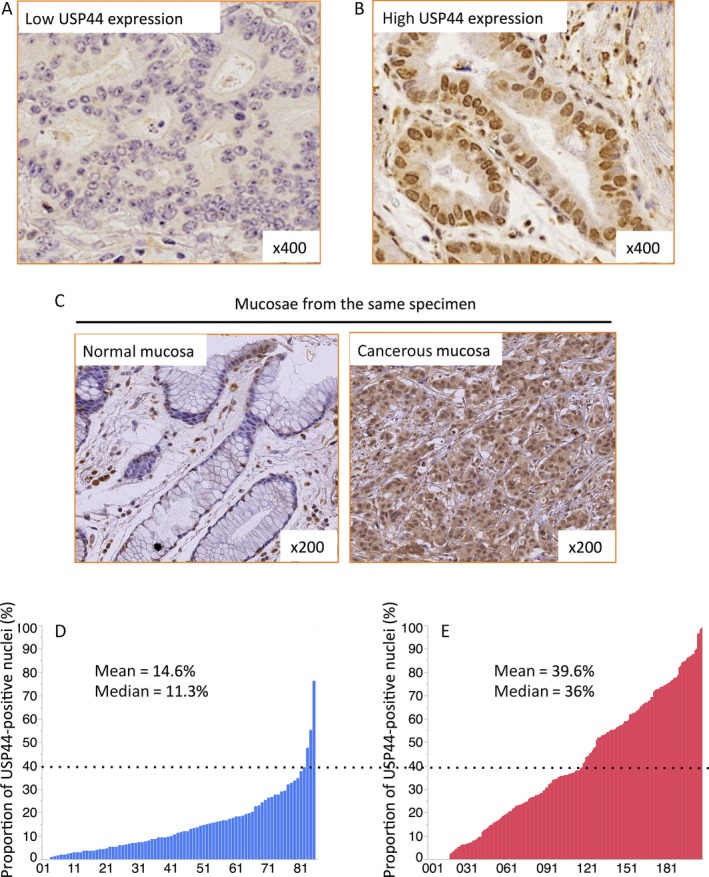
Immunohistochemistry of USP44 expression in human gastric cancer clinical samples. Immunohistochemical detection of USP44 in human gastric cancer specimens was performed. Representative low USP44 expression (A) and high USP44 expression cases (B) are shown. (C) Immunohistochemical detection of USP44 in normal mucosa (left) and cancer (right) within the same case. (D) Bar graphs showing the individual proportion of positive nuclei in the normal mucosa (*n* = 85) and (E) cancer cases (*n* = 207).

USP44 is mainly localized in the nucleus 18. No studies have yet reported criteria for evaluating USP44 expression in clinical samples using immunohistochemical staining. We evaluated all samples by three random high power fields for each sample. Evaluation was made in 100 nuclei per one field of vision, with 300 nuclei for each sample, and we counted the number of nuclei stained by the USP44 antibody. In 85 of the 207 gastric cancer samples, we were also able to observe normal mucosa, and we evaluated the normal mucosa the same as cancer tissue. We defined low USP44 expression as cases with <40% positive nuclei and high USP44 expression as cases with ≥40% positive nuclei. Representative USP low expression and USP high expression cases are shown in Figure 1A and B. A representative imaging of normal mucosa and cancer from the same specimen is shown in Figure 1C.

### Analysis for DNA ploidy

Nuclear DNA content was measured using laser scanning cytometry (LSC; CompuCyte, Westwood, MA) as described previously 19, 20. The same paraffin‐embedded blocks that were used for immunohistochemical staining were used for this analysis. A DNA content histogram was generated and DNA ploidy was determined; the DNA index (DI) was calculated according to previously published principles 21, 22. For every case, the nuclei were observed after each scan to exclude debris and attached nuclei from the analysis. The DI of G0⁄G1‐phase lymphocytes or fibroblasts were used as a reference of DI = 1.0. Tumors with a DI < 1.2 were defined as diploidy; tumors with a DI ≥ 1.2 or multi‐indexed samples were defined as aneuploidy (Fig. S1).

### High‐resolution fluorescent microsatellite analysis (HRFMA) for MSI

HRFMA has been described in detail elsewhere 23. Briefly, genomic DNA isolated from cancerous and corresponding noncancerous tissue specimens was used to amplify microsatellite loci by polymerase chain reaction (PCR) using primer sets labeled with a fluorescent compound, ROX (6‐carboxyX‐rhodamine) or HEX (6‐carboxy‐20,40,70,4,7,‐hexachrolofluorescein). The fluorescently labeled PCR products were mixed, denatured, and loaded onto an ABI 310 sequencer (Applied Biosystems, Foster City, CA) for fragment analysis. The data were processed using the GeneScan software package (Applied Biosystems). An alternation in the length of a microsatellite PCR fragment from cancerous tissues was defined as MSI positive. According to the guidelines established by the National Cancer Institute (NCI), MSI was defined by the frequency of positive findings of five reference markers: D2S123, D5S107, D10S197, D11S904, and D13S175 24. MSI status was classified as follows: microsatellite instability high (MSI‐H), >30% of loci demonstrate MSI; microsatellite instability low, ≤30% of loci demonstrate MSI; and microsatellite stability, no positive MSI detected in any of the loci. MSI‐H was labeled “MSI (+)” and the rest “MSI (−)”.

### Cell culture and materials

hTERT‐RPE1 cells were obtained from the ATCC and cultured in DMEM/F12 (Gibco) supplemented with 10% fetal bovine serum, penicillin (100 U/mL), streptomycin (100 *μ*g/mL), and hygromycin B (200 *μ*g/mL). Cells were grown in a 5% CO_2_ atmosphere at 37°C. The USP44 gene was amplified from hTERT‐RPE1 cDNA by PCR using the forward primer 5′‐CACCATGCTAGCAATGGATACGTGCAAAC‐3′ and the reverse primer 5′‐TCAGCTAAGGATTTCATTAGACGAG‐3′ and then cloned into pENTR/D‐TOPO (Thermo Fisher).

### Immunoblot

Cells were harvested and lysed in lysis buffer (20 mmol/L Tris pH 8.0, 150 mmol/L NaCl, 1 mmol/L EDTA, 0.5% NP‐40, 1 mmol/L phenylmethylsulfonyl fluoride, a protease inhibitor cocktail and a phosphatase inhibitor cocktail (Nacalai Tesque)) for 30 min on ice. Cell extracts were clarified by centrifugation, lysates were boiled in SDS loading buffer, and protein samples were separated by SDS‐PAGE. Immunoblotting was performed using the following antibodies at the indicated dilution: rabbit anti‐USP44 at 1:250 (ab205032; Abcam) and mouse anti‐*β*‐actin at 1:5000 (A5316; Sigma). Quantitative analysis was performed using the ImageQuant TL software (GE Healthcare). Images have been cropped for presentation.

### Real‐time quantitative RT‐PCR

Total RNA was isolated from cells using an RNeasy mini kit (Qiagen) according to the manufacturer's instructions. cDNA was synthesized with random primers and reverse transcriptase according to the manufacturer's instructions and the product was used for further analysis using High‐Capacity cDNA Reverse Transcription kit (Thermo Fisher). USP44 transcription was quantified using the LightCycler 480 II (Roche) PCR protocol, in which fluorescence emission attributable to binding of SYBR Green I dye to amplified products can be measured. USP44 mRNA expression levels were measured in triplicate for each sample and normalized to mRNA levels of the endogenous *β*‐actin control. The primer sequences for real‐time RT‐PCR were as follows: USP44, 5′‐CCAGTTGTACTCACAGAAGCCC‐3′ (forward) and 5′‐CCTGAATCGTTTGAGGTGCAG‐3′ (reverse) 11, and *β*‐actin, 5′‐CTGGCACCACACCTTCTACAATG‐3′ (forward) and 5′‐GGCGTACAGGGATAGCACAGC‐3′ (reverse).

### Lentiviral infection and establishment of USP44 stable cell lines

USP44 genes from pENTR/D‐TOPO and pENTR5′/EF1ap were cloned into the pcLenti6.4/R4R2/V5‐DEST vector (Gateway Technology, Thermo Fisher). Lentiviral stocks were produced using the ViraPower Lentiviral Expression System (Thermo Fisher). Three independent hTERT‐RPE1 cell lines were infected with lentivirus and selected by blasticidin (10 *μ*g/mL) to generate three cell lines with stable expression of USP44 (USP44‐1, USP44‐2, and USP44‐3).

### Chromosome spreads

Chromosome spreading was performed by GTG (G‐bands by trypsin using Giemsa) 25. Briefly, cells were treated with 0.1 *μ*g/mL colcemid for 12 h, collected and hypotonically swollen in 75 mmol/L KCl for 12 min at 37°C. Cells were fixed in Carnoy's fixative solution (75% methanol and 25% acetic acid) with three changes of the fixative. Cells were dropped onto cooled glass slides and dried at 55°C for 30 sec. Chromosomes were trypsinized and stained in 5% Giemsa for 10 min, rinsed with PBS, air‐dried and mounted.

### Statistical analysis

The statistical analysis was performed using the JMP 11.0 statistical software package (SAS Institute, Cary, NC). The Student's *t*‐test, the chi‐squared test, Fisher's exact test, and ANOVA one‐way test were used where appropriate. The Kaplan–Meier analysis was used for progression‐free survival (PFS) and overall survival (OS) using log‐rank test.

## Results

### DNA ploidy in gastric cancer

The DNA ploidy patterns of all 207 gastric cancer patients were analyzed by LSC, and 124 of the 207 total patients (60%) showed DNA aneuploidy, which was consistent with a previous report 26. We compared the DNA ploidy patterns with patient clinicopathological factors (Table S1) and found that the rate of lymphatic vessel invasion was significantly higher in DNA diploid tumors (*P* < 0.05). However, there were no significant differences in other factors, such as age, gender, histology, depth of invasion, lymph node metastasis, vascular involvement, or stage. We also compared ploidy status with MSI status (Table S2). Of the 205 total patients, only 18 (8.8%) were MSI (+). MSI (+) had no significant correlation with DNA aneuploidy.

### USP44 expression levels were higher in gastric cancer than in gastric normal mucosa

We next investigated USP44 expression by immunohistochemistry in the 207 gastric cancer samples as described in Materials and Methods. A previous study showed that USP44 localizes and functions in the nucleus 18. USP44 levels are increased in mitotic cells and rapidly decrease after cells exit from mitosis 11. There are generally very few ratios of mitotic cells. The mean values were 14.6% positive USP44 expression in normal mucosa and 39.6% in cancer (*P* < 0.0001). The proportion of positive nuclei in cancer cases was significantly higher compared with normal mucosa in the same cases (Fig. 1D and E). In addition, among the normal mucosa samples, most of the cases (82 cases, 96.5%) showed positive USP44 expression in up to 40% of nuclei, with only three cases (3.5%) showing USP44 positive expression in more than 40% of nuclei (Table S3). On the other hand, among the 207 cancer cases, 90 cases (43.5%) showed USP44‐positive expression in over 40% of nuclei. Based on these results, we defined low USP44 expression as cases with less than 40% positive USP44 nuclei staining and high USP44 expression as cases with more than 40% positive USP44 nuclei staining.

### Strong correlation between high USP44 expression and DNA aneuploidy in gastric cancer

High USP44 expression was observed in 90 (43.5%) of the 207 patients. While we did not observe a significant correlation between clinicopathological features and USP44 expression, we found that high USP44 expression strongly correlated with DNA aneuploidy in gastric cancer (Table 1) (*P* < 0.001). This finding suggests that USP44 expression level seemed to be important factor for DNA aneuploidy in gastric cancer. Thus, we performed further analyses and subdivided the entire patient group into diploid and aneuploidy groups (Table 2). In the diploid group, there was no significant clinicopathological factor correlated with USP44 expression. However, in the aneuploidy group, tumor invasion was significantly deeper (*P* < 0.01) and distant metastasis rate tended to be higher in the high USP44 expression group.

**Table 1:** USP44 expression and clinicopathological factors in gastric cancer

Factors	USP44 expression		
	Low (*n* = 117)	High (*n* = 90)	*P*‐values
Age (mean ± SD)	62.8 ± 11.5	64.8 ± 12.7	0.25
Sex			
Male	78 (66.7)	60 (66.7)	1
Female	39 (33.3)	30 (33.3)	
Differentiation			
Well/mod	52 (44.4)	35 (38.9)	0.62
Poor/sig	57 (48.7)	50 (55.6)	
Other	8 (6.8)	5 (5.6)	
Depth of invasion			
M, SM, MP	34 (29.1)	16 (17.8)	0.06
SS, SE, SI	83 (70.9)	74 (82.2)	
Lymph node metastasis			
Negative	38 (32.5)	27 (30)	0.55
Positive	79 (67.5)	63 (70)	
Vascular involvement			
Negative	69 (59)	50 (55.6)	0.62
Positive	48 (41)	40 (42.5)	
Lymphatic vessel invasion			
Negative	28 (23.9)	31 (34.4)	0.1
Positive	89 (76.1)	59 (65.6)	
Distant metastasis			
Negative	94 (80.3)	66 (73.3)	0.25
Positive	23 (19.4)	24 (26.7)	
Stage			
I	23 (19.7)	12 (13.3)	0.44
II	28 (23.9)	20 (22.2)	
III	43 (36.8)	33 (36.7)	
IV	23 (19.7)	25 (27.8)	
DNA ploidy			
Diploidy	59 (49.6)	24 (26.7)	0.0005^a^
Aneuploidy	58 (50.4)	66 (73.3)	

Values in parentheses indicate %.

Well, well differentiated carcinoma; mod, moderately differentiated carcinoma; poor, poorly differentiated carcinoma; sig, signet‐ring cell carcinoma; M, mucosa; SM, submucosa: MP, muscularis propria; SS, subserosa; SE, penetration of serosa; SI, invasion of adjacent structures.

a *P* < 0.001.

**Table 2:** Subgroup analysis for USP44 expression and clinicopathological factors

Factors	USP44 expression		
	Low	High	*P*‐values
Diploid cases	(*n* = 59)	(*n* = 24)	
Age (mean ± SD)	61.8 ± 13	62.8 ± 14.8	0.77
Sex			
Male	42 (71.2)	14 (58.3)	0.26
Female	17 (28.8)	10 (41.7)	
Differentiation			
Well/mod	22 (37.3)	8 (33.3)	0.8
Poor/sig	32 (54.2)	15 (62.5)	
Other	5 (8.5)	1 (4.2)	
Depth of invasion			
M, SM, MP	15 (25.4)	5 (20.8)	0.78
SS, SE, SI	44 (74.6)	19 (79.2)	
Lymph node metastasis			
Negative	20 (33.9)	10 (41.7)	0.5
Positive	39 (66.1)	14 (58.3)	
Vascular involvement			
Negative	36 (61)	16 (66.7)	0.63
Positive	23 (39)	8 (33.3)	
Lymphatic vessel invasion			
Negative	11 (18.6)	7 (29.2)	0.29
Positive	48 (81.4)	17 (70.8)	
Distant metastasis			
Negative	45 (76.3)	20 (83.3)	0.57
Positive	14 (23.7)	4 (16.7)	
Stage			
I	10 (17)	3 (12.5)	0.83
II	15 (25.4)	8 (33.3)	
III	20 (33.9)	9 (37.5)	
IV	14 (23.7)	4 (16.7)	
Aneuploid cases	(*n* = 58)	(*n* = 66)	
Age (mean ± SD)	64 ± 13	65.4 ± 11.9	0.48
Sex			
Male	36 (62.1)	46 (69.7)	0.37
Female	22 (37.9)	20 (30.3)	
Differentiation			
Well/mod	30 (51.7)	27 (40.9)	0.49
Poor/sig	25 (43.1)	35 (53)	
Other	3 (5.2)	4 (6.1)	
Depth of invasion			
M, SM, MP	19 (32.8)	11 (16.7)	0.0036^a^
SS, SE, SI	39 (67.2)	55 (83.3)	
Lymph node metastasis			
Negative	18 (31)	17 (25.8)	0.51
Positive	40 (69)	49 (74.2)	
Vascular involvement			
Negative	33 (56.9)	34 (51.5)	0.55
Positive	25 (43.1)	32 (48.5)	
Lymphatic vessel invasion			
Negative	17 (29.3)	24 (36.3)	0.4
Positive	41 (70.7)	42 (63.6)	
Distant metastasis			
Negative	49 (84.5)	46 (69.7)	0.05
Positive	9 (15.5)	20 (30.3)	
Stage			
I	13 (22.4)	9 (13.6)	0.16
II	13 (22.4)	12 (18.2)	
III	23 (39.7)	24 (36.4)	
IV	9 (15.5)	21 (31.8)	

Values in parentheses indicate %.

Well, well differentiated carcinoma; mod, moderately differentiated carcinoma; poor, poorly differentiated carcinoma; sig, signet‐ring cell carcinoma; M, mucosa; SM, submucosa: MP, muscularis propria; SS, subserosa; SE, penetration of serosa; SI, invasion of adjacent structures.

a *P* < 0.05.

### The combination analysis of DNA ploidy status and USP44 expression supplies useful prognostic information in gastric cancer

**Figure 2:**
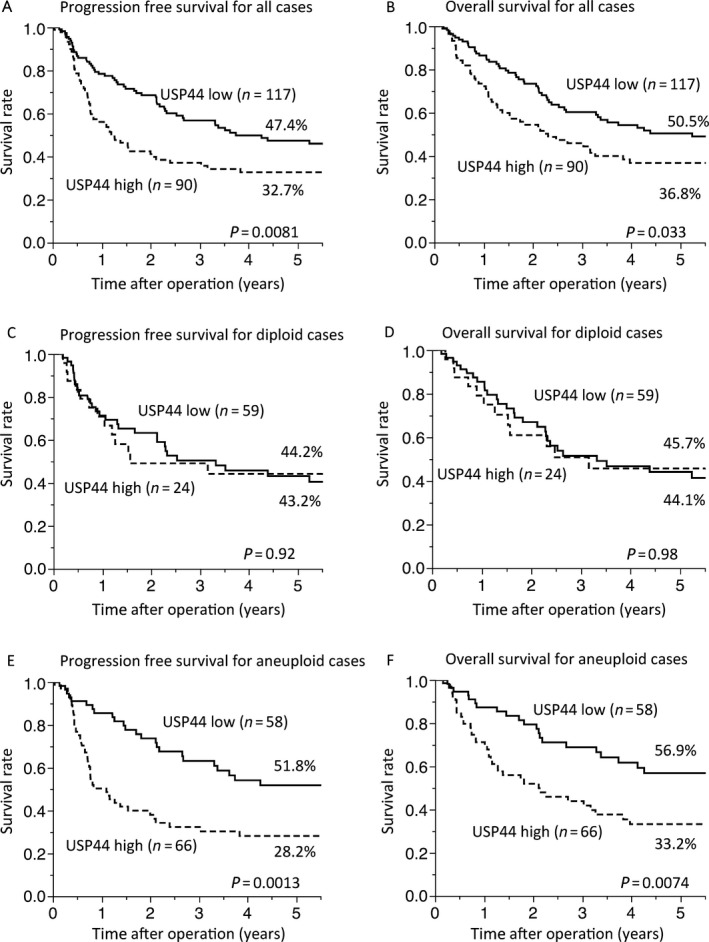
Kaplan–Meier curves for gastric cancer cases separated by USP44 expression. (A) Progression‐free survival (PFS) and (B) overall survival (OS) curves for the low USP44 expression group (solid line) and high USP44 expression group (dotted line) (all cases, *n* = 207). (C) PFS and (D) OS in subgroups according to USP44 expression among diploid cases (*n* = 83). (E) PFS and (E) OS in subgroups according to USP44 expression among aneuploid cases (*n* = 124). *P*‐value was calculated using the log‐rank test.

We next performed survival time analysis of the gastric cancer cases. DNA ploidy status had no influence on prognosis in this study (Fig. S2A and B). We performed further subgroup analyses by dividing cases according to USP44 expression. There was no significant difference in survival rates between diploidy and aneuploidy (Fig. S2C and D). The 5‐year PFS and OS rates of the high‐USP44 expression group were significantly poorer than those in the low‐USP44 expression group (log‐rank, *P* < 0.05) (Fig. 2A and B). We next performed subgroup analysis and divided patients into diploid and aneuploidy groups to clarify the influence of high USP44 expression on DNA ploidy status. Interestingly, in the diploid group, we observed no difference in survival rates between the low USP44 and high USP44 groups (Fig. 2C and D). However, in the aneuploid group, high USP44 cases had remarkable poor prognosis and low‐USP44 expression cases had good prognosis (Fig. 2E and F). These results indicated that high USP44 expression seemed to be a prognostic factor for gastric cancer with CIN.

To determine independent prognostic factors, we performed univariate and multivariate analyses with the Cox proportional hazard model in terms of PFS (Table S5) and OS (Table 3). If the *P*‐value of a factor was <0.1 in the univariate analyses, we included that factor in the multivariate analyses. Results from the multivariate analyses showed that USP44 was an independent poor prognostic factor of PFS for all gastric cancer patients. In subgroup analyses, high USP44 expression was an independent poor prognostic factor of PFS and OS in aneuploidy gastric cancer, but not in diploidy cases.

**Table 3:** Univariate and multivariate analyses for overall survival

Factor	Univariate analysis		Multivariate analysis	
	HR (95% CI)	*P*	HR (95% CI)	*P*
All cases (*n* = 207)				
Age ≥70 years (vs. <70 years)	1.66 (1.11–2.46)	0.0134	1.95 (1.28–2.95)	0.0021
Female (vs. male)	1.18 (0.78–1.76)	0.4220	−	−
pT3,4 (vs. pT1,2)	2.97 (1.69–5.71)	<0.0001	2.23 (1.18–4.54)	0.0122
pN+ (vs. pN−)	3.26 (2.02–5.53)	<0.0001	2.37 (1.40–4.20)	0.0011
pM+ (vs. pM−)	2.89 (1.91–4.32)	<0.0001	1.70 (1.09–2.63)	0.0202
Aneuploid (vs. diploid)	0.97 (0.66–1.45)	0.8896	−	−
high USP44 (vs. low)	1.51 (1.03–2.23)	0.0354	1.36 (0.91–2.02)	0.1359
Diploid cases (*n* = 83)				
Age ≥ 70 years (vs. <70 years)	1.44 (0.75–2.68)	0.2685	−	−
Female (vs. male)	1.72 (0.90–3.18)	0.0959	1.53 (0.79–2.86)	0.2021
pT3,4 (vs. pT1,2)	2.72 (1.17–7.92)	0.0172	1.74 (0.71–5.22)	0.2397
pN+ (vs. pN−)	2.45 (1.27–5.12)	0.0068	1.82 (0.89–3.94)	0.1006
pM+ (vs. pM−)	3.71 (1.90–7.03)	0.0002	2.54 (1.25–5.12)	0.0108
high USP44 (vs. low)	1.01 (0.51–1.90)	0.9750	−	−
Aneuploid cases (*n* = 124)				
Age ≥70 years (vs. <70 years)	1.88 (1.12–3.12)	0.0174	1.99 (1.15–3.41)	0.0141
Female (vs. male)	0.93 (0.53–1.58)	0.7995	−	−
pT3,4 (vs. pT1,2)	3.08 (1.50–7.43)	0.0014	2.00 (0.86–5.31)	0.1128
pN+ (vs. pN−)	4.35 (2.18–9.94)	<0.0001	3.50 (1.59–8.63)	0.0014
pM+ (vs. pM−)	2.60 (1.50–4.38)	0.0009	1.17 (0.64–2.12)	0.5971
high USP44 (vs low)	2.01 (1.20‐3.42)	0.0075	1.83 (1.04‐3.29)	0.0357

CI, confidence interval; HR, hazard ratio; USP44, ubiquitin‐specific protease 44.

### Overexpression of USP44 leads to CIN

**Figure 3:**
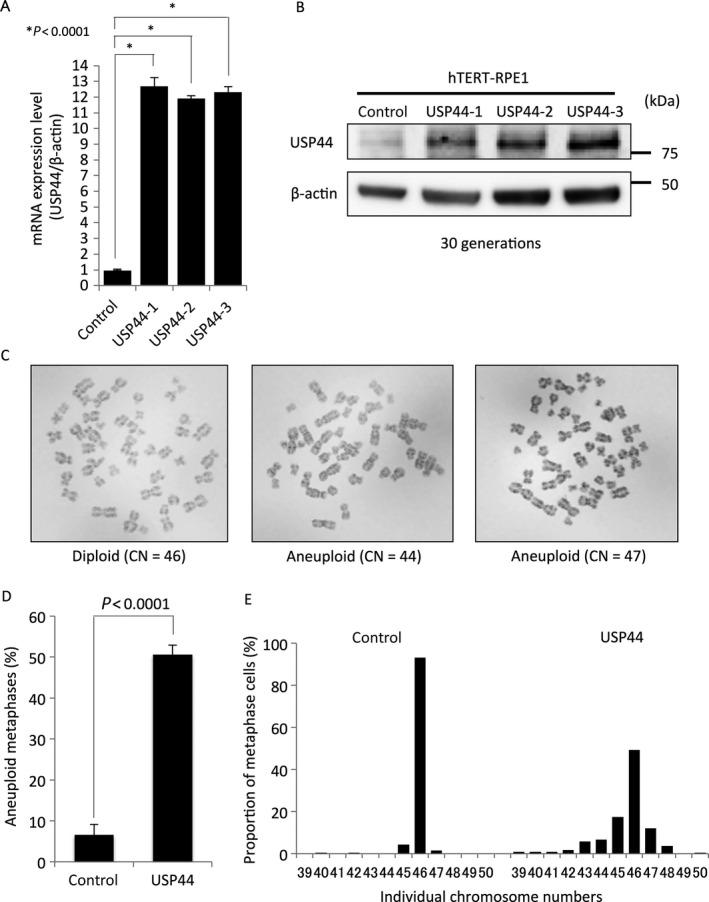
Overexpression of USP44 leads to aneuploidy. (A) USP44 mRNA expression levels in control hTERT‐RPE1 (*n* = 9), USP44‐1 (*n* = 3), USP44‐2 (*n* = 3), and USP44‐3 (*n* = 3) cell lines were measured using qRT‐PCR and standardized by *β*‐actin mRNA levels. *P < 0.0001. (B) Western blot analysis of USP44 protein expression in control hTERT‐RPE1 cells and the three USP44 stably overexpressing cells was performed after 30 generations. *β*‐actin served as loading control. (C) Representative images of chromosomes of single cell samples were shown. Diploidy (left) and aneuploidy (middle, loss; right, gain) are shown. (D) Proportion of cells with aneuploidy was determined after 30 generations in control and USP44 overexpression cells. Data are means ± SD from three independent experiments (>50 cells per experiment). (E) Individual chromosome numbers from chromosome spreads were determined (total cell number: control, *n* = 204; USP44, *n* = 223). The normal chromosome number of hTERT‐RPE1 cells is 46.

Finally, we investigated whether high USP44 expression caused CIN. A previous study reported that overexpression of USP44 in mouse embryonic fibroblasts induced CIN 13. However, no studies have examined its effects in human cell lines. To address this question, we established three hTERT‐RPE1 cell lines stably expressing USP44 (RPE1‐USP44) using a lentiviral vector and confirmed upregulation of USP44 mRNA and protein levels (Fig. 3A and B). We cultured control RPE1 cells and RPE1‐USP44 cells for 30 generations and performed chromosome spreading and chromosome counts (Fig. 3C). We found that the proportion of aneuploidy cells was significantly increased in RPE1‐USP44 cells (50.6 ± 2.3%) compared with controls (6.6 ± 2.49%) (*P* < 0.0001) (Fig. 3D and E). These results indicate that stable overexpression of USP44 leads to CIN in a human cell line.

## Discussion

In this study, we found that USP44 expression was higher in gastric cancer than in gastric normal mucosa and showed that USP44 overexpression related to DNA aneuploidy in gastric cancer. We also found that high USP44 expression was an independent poor prognostic factor for gastric cancer with DNA aneuploidy. Furthermore, we demonstrated that overexpression of USP44 induced DNA aneuploidy in a human cell line.

USP44 exhibits at least two cellular functions: one is the regulation of SAC proteins to prevent premature anaphase onset by deubiquitinating the Cdc20‐Mad2 complex 11; and the second is the control of centrosome positioning in metaphase to prevent DNA aneuploidy by forming a complex with centrin 12. Both of these functions are required to prevent chromosome mis‐segregation. USP44 is an important regulator in mitosis, and therefore suppression or overexpression of USP44 leads to aneuploidy, resulting in an induction of CIN. Previous studies reported that downregulation of USP44 leads to chromosome mis‐segregation and results in CIN 12, 14, 15. On the other hand, Zhang et al. reported that the overexpression of USP44 leads to an increase in mitotic errors and aneuploidy in murine embryonic fibroblasts 13. We evaluated the proportion of USP44‐positive nuclei in clinical specimens of gastric cancer and normal mucosa by immunohistochemical staining and found that USP44 expression was significantly higher in gastric cancer than normal mucosa (Fig. 1). This result indicated that abnormally high USP44 expression relates to gastric cancer pathophysiology. Furthermore, we confirmed a significant correlation of high USP44 expression and DNA aneuploidy in gastric cancer. To elucidate the consequence of the relationship between high USP44 expression and DNA aneuploidy, we verified for the first time that USP44 overexpression in RPE1‐USP44 stable cells frequently led to aneuploidy (gain or loss of chromosome number) compared with control cells (Fig. 3). This result shows for the first time that USP44 overexpression can lead to DNA aneuploidy in human cells. A previous study showed that abnormally elevated USP44 excessively acts on the Cdc20‐Mad2 complex, and prolongs the inactivation of the Mad2‐Cdc20 complex 13. Hence, APC/C cannot be activated and cut cohesin with the appropriate timing. As a result, mitotic exit is delayed and mis‐segregation, such as lagging chromosomes and chromosome disjunction, develops. This mis‐segregation causes DNA aneuploidy in each cell. Indeed, the growth of RPE1‐USP44 cells was significantly slower than control cells, and this might be due to the USP44 overexpression‐induced mitotic delay (Fig. S3). Together our results show that USP44 overexpression induces DNA aneuploidy in gastric cancer.

Aneuploidy is a hallmark of cancer cells and relates to tumorigenesis, tumor progression, and prognosis 4. However, the mechanisms and significance of aneuploidy in cancer are not yet clear 27. In gastric cancer, the correlation of aneuploidy with prognosis remains controversial 28. A mouse model in which chromosome mis‐segregation was induced by inactivation of a component of the chromosome segregation machinery, CENP‐E, indicated that aneuploidy acts as an oncogenic factor in some cell types but inhibits tumorigenesis in others 29. Random aneuploidy caused by transient overexpression of Mad2 in the mouse initiates tumor formation only in certain cell types 30. A mouse model expressing a hypomorphic allele of BubR1 displays progressive aneuploidy and exhibits an accelerated aging phenotype but without increased incidence of tumorigenesis 31. Although monosomy of chromosome 21 increases tumor number, trisomy of chromosome 21 reduces tumor number in the colon cancer APC*Min* mouse model 32. Collectively, these results suggest that aneuploidy may behave as both a tumor suppressor and tumor promoter. In our study, the status of DNA aneuploidy alone was not a prognostic factor in gastric cancer. DNA aneuploidy is complex and includes various phenotypes. Because the various patterns of aneuploidy were mixed in our clinical samples, there was no prognostic difference between euploidy and aneuploidy among all cases. In fact, the data presented by TCGA Research Network indicated that there were no significant differences in survival or recurrence rate between CIN and GS in gastric adenocarcinoma 1.

While aneuploidy was not shown to be a prognostic factor in gastric cancer, our findings showed that the high‐USP44 expression group was associated with a poorer prognosis than the low expression group. We also subdivided all cases into diploidy and aneuploidy to perform subgroup analysis by USP44 expression level. Importantly, although there was no significant prognostic difference in USP44 expression in the diploid group, high USP44 expression was an independent poor prognostic factor in the aneuploid group. High USP44 expression also correlated with depth of invasion and distant metastasis only in the aneuploid group. In the diploid group, there was no correlation between USP44 expression and clinicopathological factors. Our data suggest that aneuploidy caused by USP44 overexpression promotes tumor progression in gastric cancer. When we subdivided the cases into high USP44 expression and low expression groups, DNA ploidy status have some influences for overall survivals, but have no significant differences. This means that the USP44 and aneuploidy may have quantitative interaction.

Deubiquitinating enzymes have various functions and thus influences normal and cancer cells through their regulation of protein levels. Previous reports showed that USP22, another ubiquitin‐specific protease, promotes tumor invasion and metastasis 33, 34. In addition, other reports showed that a molecule related to aneuploidy induces the invasion of cancer cells 35. USP44 is usually antagonized ubiquitinating enzymes such as UbcH10. We think that on the diploidy and high USP44 cases, such ubiquitinating enzymes may antagonize USP44 action 11. USP44 likely has novel functions that have not yet been discovered; overexpression of these unknown functions may also affect tumor invasion or pro‐metastasis activities in gastric cancer. We speculate that the unknown functions of USP44 and DNA aneuploidy may have synergistic progressive effects on cancer invasion and metastasis. To clarify the mechanism and biological function of USP44 in cancer, further experiments and functional analyses of USP44 are required.

In summary, here we report that the combination of USP44 expression and DNA ploidy status might serve as an independent prognostic marker in gastric cancer. The combination analysis of DNA ploidy status and other factors is a useful clinically applicable method to provide detailed information 36, 37. Although further experiments will be required to determine the functions of USP44 and its role within cancer, our findings will be helpful for clinical molecular classification.

## Conflict of Interest

The authors declare no potential conflicts of interest.

## Supporting information


**Figure S1.** Analyses of DNA ploidy in gastric cancer cases by laser scanning cytometry. DNA ploidy was evaluated in all gastric cancer cases using laser scanning cytometry. Representative figures of DNA content measurements of each cell for a diploid case (A) and aneuploid case (B) are shown. In (A), only two peaks for G0/G1 cells (diploidy =1.00) and S/G2 cells (tetraploidy = 1.98) were detected. In (B), an abnormal peak (=1.62) caused by aneuploidy in cancer cells was detected.

**Figure S2.** Kaplan–Meier curves for gastric cancer patients separated by DNA ploidy status. (A) Progression‐free survival (PFS) and (B) overall survival (OS) curves for all cases (*n* = 207) according to diploid cases (blue line) and aneuploid cases (red line). There were no significant differences in 5‐year PFS and OS between diploidy and aneuploidy. Overall survival curves in the (C) USP44 low subgroup and (D) USP44 high subgroup according to diploid cases (blue line) and aneuploid cases (red line). *P*‐value was calculated using the log‐rank test.

**Figure S3.** Cell growth curve of control RPE1 and stable RPE1‐USP44‐1 cells. Three independent experiments were performed for each cell line (control RPE1 and RPE1‐USP44). Six 60‐mm dishes were prepared for each experiment, and 1.0 × 10^5^ cells were seeded in each dish. Cells were harvested at 24 h, 48 h, 72 h, 96 h, 120 h, and 144 h and counted using a cell counter. Upon cell confluence, cells were harvested and replated.

**Table S1.** DNA ploidy and clinicopathological factors of gastric cancer cases.

**Table S2.** DNA ploidy and MSI status in gastric cancer.

**Table S3.** Comparison of USP44 expression between normal mucosa and cancer tissue.

**Table S4.** Subgroup analysis for DNA ploidy status and clinicopathological factors.

**Table S5.** Univariate and multivariate analyses for progression‐free survival.
