# Supplementary figures and images for: High ubiquitin‐specific protease 44 expression induces DNA aneuploidy and provides independent prognostic information in gastric cancer
Source: Cancer Med. 2017 May 23;6(6):1453–64. doi: 10.1002/cam4.1090 (PMC5463085; doi:10.1002/cam4.1090)

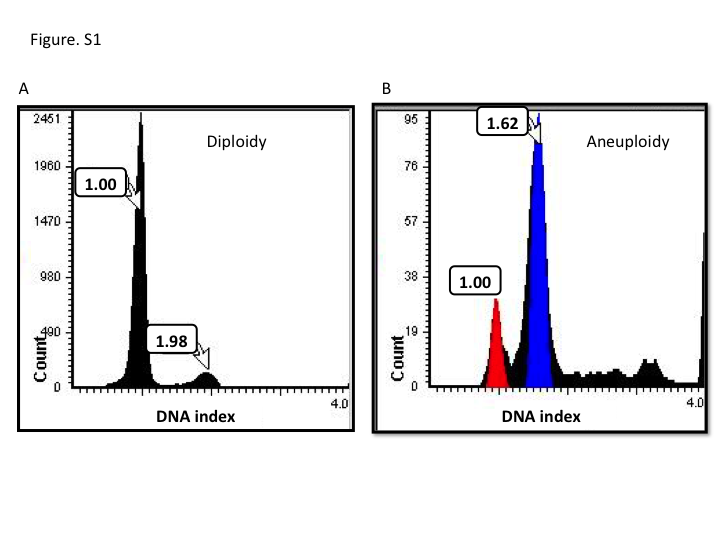

Supplement: Supplementary file 1 — Figure S1. Analyses of DNA ploidy in gastric cancer cases by laser scanning cytometry. DNA ploidy was evaluated in all gastric cancer cases using laser scanning cytometry. Representative figures of DNA content measurements of each cell for a diploid case (A) and aneuploid case (B) are shown. In (A), only two peaks for G0/G1 cells (diploidy =1.00) and S/G2 cells (tetraploidy = 1.98) were detected. In (B), an abnormal peak (=1.62) caused by aneuploidy in cancer cells was detected. [file CAM4-6-1453-s001.tiff]

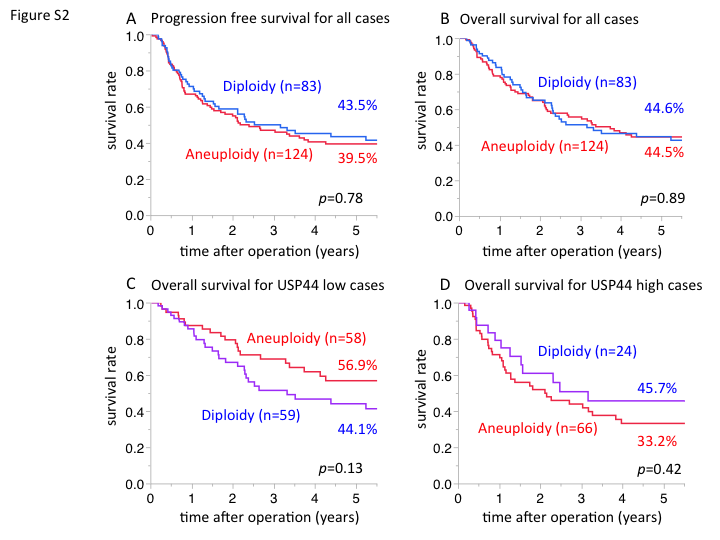

Supplement: Supplementary file 2 — Figure S2. Kaplan–Meier curves for gastric cancer patients separated by DNA ploidy status. (A) Progression‐free survival (PFS) and (B) overall survival (OS) curves for all cases (n = 207) according to diploid cases (blue line) and aneuploid cases (red line). There were no significant differences in 5‐year PFS and OS between diploidy and aneuploidy. Overall survival curves in the (C) USP44 low subgroup and (D) USP44 high subgroup according to diploid cases (blue line) and aneuploid cases (red line). P‐value was calculated using the log‐rank test. [file CAM4-6-1453-s002.tiff]

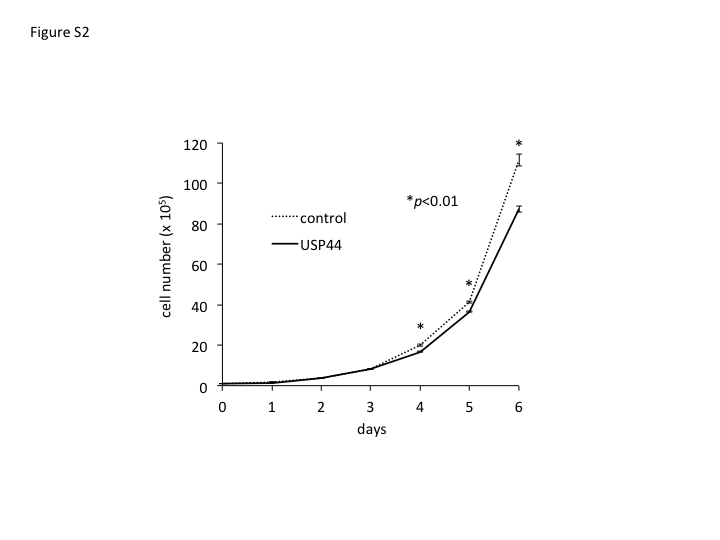

Supplement: Supplementary file 3 — Figure S3. Cell growth curve of control RPE1 and stable RPE1‐USP44‐1 cells. Three independent experiments were performed for each cell line (control RPE1 and RPE1‐USP44). Six 60‐mm dishes were prepared for each experiment, and 1.0 × 10^5^ cells were seeded in each dish. Cells were harvested at 24 h, 48 h, 72 h, 96 h, 120 h, and 144 h and counted using a cell counter. Upon cell confluence, cells were harvested and replated. [file CAM4-6-1453-s003.tiff]
