# Supplementary material for: High ubiquitin‐specific protease 44 expression induces DNA aneuploidy and provides independent prognostic information in gastric cancer
Source: Cancer Med. 2017 May 23;6(6):1453–64. doi: 10.1002/cam4.1090 (PMC5463085; doi:10.1002/cam4.1090)
Supplement: Supplementary file 4 — Table S1. DNA ploidy and clinicopathological factors of gastric cancer cases. [file CAM4-6-1453-s004.doc]

Table S1. DNA ploidy and clinicopathological factors of gastric cancer cases

DNA ploidy

Factors Diploidy (n=83) Aneuploidy (n=124) P-values

Age (mean ± SD) 62.1±13.5 64.8±10.9 0.11

Sex

Male 56 (67.5) 82 (65.6) 0.88

Female 27 (32.5) 42 (34.4)

Differentiation

Well/Mod 30 (16.1) 57 (46) 0.36

Por/Sig 47 (56.6) 60 (48.4)

Other 6 (7.2) 7 (5.6)

Depth of invasion

M, SM, MP 20 (24.1) 30 (24.2) 0.99

SS, SE, SI 63 (75.9) 94 (75.8)

Lymph node metastasis

Negative 30 (36.1) 35 (28.2) 0.28

Positive 53 (63.9) 89 (71.8)

Vascular involvement

Negative 51 (61.4) 68 (54.8) 0.39

Positive 32 (38.6) 56 (45.2)

Lymphatic vessel invasion

Negative 17 (20.5) 42 (33.9) 0.042*

Positive 66 (79.5) 82 (66.1)

Distant metastasis

Negative 65 (78.3) 95 (76.6) 0.87

Positive 18 (21.7) 29 (23.4)

Stage

I 13 (15.7) 22 (17.7) 0.56

II 24 (28.9) 25 (20.2)

III 28 (33.7) 47 (37.9)

IV 18 (21.7) 30 (24.2)

Values in parentheses indicate %. **P* < 0.05.

Well, well differentiated carcinoma; mod, moderately differentiated carcinoma; poor, poorly differentiated carcinoma; sig, signet-ring cell carcinoma.

M, mucosa; SM, submucosa: MP, muscularis propria; SS, subserosa; SE, penetration of serosa; SI, invasion of adjacent structures.
