# Supplementary material for: High ubiquitin‐specific protease 44 expression induces DNA aneuploidy and provides independent prognostic information in gastric cancer
Source: Cancer Med. 2017 May 23;6(6):1453–64. doi: 10.1002/cam4.1090 (PMC5463085; doi:10.1002/cam4.1090)
Supplement: Supplementary file 5 — Table S2. DNA ploidy and MSI status in gastric cancer. [file CAM4-6-1453-s005.doc]

Table S2. DNA ploidy and MSI status in gastric cancer.

DNA ploidy

MSI status Diploidy Aneuploidy P-values

MSI (-) 75(91.5) 112(91.1) 1

MSI (+) 7(8.5) 11(8.9)

Values in parentheses indicate %.

MSI, microsatellite instability.
