# Supplementary material for: High ubiquitin‐specific protease 44 expression induces DNA aneuploidy and provides independent prognostic information in gastric cancer
Source: Cancer Med. 2017 May 23;6(6):1453–64. doi: 10.1002/cam4.1090 (PMC5463085; doi:10.1002/cam4.1090)
Supplement: Supplementary file 6 — Table S3. Comparison of USP44 expression between normal mucosa and cancer tissue. [file CAM4-6-1453-s006.doc]

Table S3. Comparison of USP44 expression between normal mucosa and cancer tissue

proportion of normal mucosa cancer tissue

stained nucleus (%) (n=85) (n=207)

0-20 65 (76.5) 61 (29.4)

21-40 17 (20) 56 (27.1)

41-60 2 (2.4) 38 (18.4)

61-80 1 (1.2) 36 (17.4)

81-100 0 (0) 16 (7.7)

Values in parentheses indicate %.
