# Supplementary material for: High ubiquitin‐specific protease 44 expression induces DNA aneuploidy and provides independent prognostic information in gastric cancer
Source: Cancer Med. 2017 May 23;6(6):1453–64. doi: 10.1002/cam4.1090 (PMC5463085; doi:10.1002/cam4.1090)
Supplement: Supplementary file 7 — Table S4. Subgroup analysis for DNA ploidy status and clinicopathological factors. [file CAM4-6-1453-s007.doc]

Table S4. Subgroup analysis for DNA ploidy status and clinicopathological factors

DNA ploidy status

Factors Diploidy Aneuploidy P-values

USP44 low cases (n=59) (n=58)

Age (mean ± SD) 61.8±13 64±9.8 0.3

Sex

Male 42 (71.2) 36 (62.1) 0.3

Female 17 (28.8) 22 (37.9)

Differentiation

Well/Mod 22 (37.3) 30 (51.7) 0.28

Poor/Sig 32 (54.2) 25 (43.1)

Other 5 (8.5) 3 (5.2)

Depth of invasion

M, SM, MP 15 (25.4) 19 (32.8) 0.38

SS, SE, SI 44 (74.6) 39 (67.2)

Lymph node metastasis

Negative 20 (33.9) 18 (31) 0.74

Positive 39 (66.1) 40 (69)

Distant metastasis

Negative 45 (76.3) 49 (84.5) 0.26

Positive 14 (23.7) 9 (15.5)

Stage

I, II 25 (42.4) 26 (44.8) 0.79

III, IV 34 (57.6) 32 (55.2)

USP44 high cases (n=24) (n=66)

Age (mean ± SD) 62.8±14.8 65.4±11.9 0.38

Sex

Male 14 (58.3) 46 (69.7) 0.31

Female 10 (41.7) 20 (30.3)

Differentiation

Well/ Mod 8 (33.3) 27 (40.9) 0.72

Poor/ Sig 15 (62.5) 35 (53)

Other 1 (4.2) 4 (6.1)

Depth of invasion

M, SM, MP 5 (20.8) 11 (16.7) 0.65

SS, SE, SI 19 (79.2) 55 (83.3)

Lymph node metastasis

Negative 10 (41.7) 17 (25.8) 0.15

Positive 15 (58.3) 49 (74.2)

Distant metastasis

Negative 20 (83.3) 46 (69.7) 0.2

Positive 4 (16.7) 20 (30.3)

Stage

I, II 11 (45.8) 21 (31.8) 0.22

III, IV 13 (54.2) 45 (68.2)

Values in parentheses indicate %.

Well, well differentiated carcinoma; mod, moderately differentiated carcinoma; poor, poorly differentiated carcinoma; sig, signet-ring cell carcinoma.

M, mucosa; SM, submucosa: MP, muscularis propria; SS, subserosa; SE, penetration of serosa; SI, invasion of adjacent structures.
