# Supplementary material for: High ubiquitin‐specific protease 44 expression induces DNA aneuploidy and provides independent prognostic information in gastric cancer
Source: Cancer Med. 2017 May 23;6(6):1453–64. doi: 10.1002/cam4.1090 (PMC5463085; doi:10.1002/cam4.1090)
Supplement: Supplementary file 8 — Table S5. Univariate and multivariate analyses for progression‐free survival. [file CAM4-6-1453-s008.doc]

Table S5. Univariate and multivariate analyses for progression free survival

Univariate Analysis Multivariate Analysis

Factor HR (95% CI) P HR (95% CI) P

All cases (n=207)

Age ≥70 y (vs <70 y) 1.48 (1.00-2.15) 0.0475 1.88 (1.26-2.78) 0.0024

Female (vs Male) 1.25 (0.84-1.82) 0.2691 − −

pT3,4 (vs pT1,2) 3.24 (1.89-6.06) <0.0001 2.22 (1.21-4.37) 0.0088

pN+ (vs pN-) 3.23 (2.05-5.33) <0.0001 2.24 (1.36-3.84) 0.0012

pM+ (vs pM-) 3.69 (2.49-5.40) <0.0001 2.40 (1.58-3.61) <0.0001

Aneuploid (vs Diploid) 1.05 (0.73-1.54) 0.7767 − −

USP44 high (vs low) 1.63 (1.13-2.36) 0.0091 1.52 (1.04-2.22) 0.0290

Diploid cases (n=83)

Age ≥70 y (vs <70 y) 1.20 (0.64-2.20) 0.5553 − −

Female (vs Male) 2.05 (1.11-3.70) 0.0223 2.02 (1.09-3.70) 0.0271

pT3,4 (vs pT1,2) 3.14 (1.36-9.10) 0.0052 1.87 (0.77-5.58) 0.0295

pN+ (vs pN-) 2.36 (1.25-4.76) 0.0072 1.77 (0.91-3.68) 0.0962

pM+ (vs pM-) 4.63 (2.47-8.51) <0.0001 3.46 (1.79-6.60) 0.0003

USP44 high (vs low) 1.03 (0.53-1.90) 0.9224 − −

Aneuploid cases(n=124)

Age ≥70 y (vs <70 y) 1.74 (1.05-2.80) 0.0297 2.18 (1.29-3.63) 0.0039

Female (vs Male) 0.91 (0.53-1.49) 0.7031 − −

pT3,4 (vs pT1,2) 3.27 (1.65-7.42) 0.0003 2.10 (0.95-5.20) 0.0691

pN+ (vs pN-) 4.34 (2.26-9.42) <0.0001 3.21 (1.52-7.47) 0.0016

pM+ (vs pM-) 3.31 (1.97-5.42) <0.0001 1.76 (1.01-3.05) 0.0474

USP44 high (vs low) 2.19 (1.35-3.63) 0.0014 1.90 (1.12-3.29) 0.0168

CI, confidence interval; HR, hazard ratio; USP44, ubiquitin-specific protease 44.
